# Less cost by using hanging maneuver and Pringle maneuver in left lateral hepatectomy through small laparotomy wound—experience of Southern Taiwan

**DOI:** 10.1186/s12957-015-0764-6

**Published:** 2016-01-08

**Authors:** Ting-Lung Lin, Ruslan Alikhanov, Sheng-Chih Kuo, Wei-Feng Li, Chao-Long Chen, Shih-Ho Wang, Chih-Che Lin, Yueh-Wei Liu, Chee-Chien Yong, Yu-Hung Lin, Chih-Chi Wang

**Affiliations:** Division of General Surgery, Department of Surgery, Kaohsiung Chang Gung Memorial Hospital, Chang Gung University College of Medicine, 123 Ta-Pei Road, Niao-Sung, Kaohsiung 833 Taiwan

**Keywords:** Minimally invasive surgery, Hepatectomy, Laparoscopy, Left lateral segment

## Abstract

**Background:**

Laparoscopic segmentectomy for liver tumor located in the left lateral segment (LLS) is thought to be a standard protocol nowadays with several advantages, such as small wound, few blood loss, and short hospital stay. However, there are still many disadvantages during executing laparoscopic LLS segmentectomy. This manuscript aims to present the technique to execute LLS segmentectomy with small incision, hanging maneuver without Pringle maneuver in patients with tumor at LLS of the liver.

**Material and methods:**

Between November 2010 and July 2011, hepatectomies through small incision for nine patients with benign and malignant tumors were performed at Kaohsiung Chang Gung Memorial Hospital, Taiwan. Perioperative and postoperative results, such as operation time, blood loss, incisional width, and postoperative stay were used to determine consequents for this technique.

**Result:**

Results demonstrated that modified LLS segmentectomy by the author’s team was performed successfully in patient with liver tumor with fewer blood loss, smaller incisional width, and lower hospital cost than traditional open surgery. In addition, the instrument cost and blood loss in our series were less than that in laparoscopic LLS segmentectomy in published literature.

**Conclusion:**

Authors concluded that minimally incisional segmentectomy, with less cost and technical demanding, could be an alternative choice in patient with liver tumor at LLS.

## Introduction

Hepatectomy is the standard treatment for many benign and malignant liver diseases. Traditional open surgery has been performed worldwide for decades. Laparoscopic hepatectomy becomes a standard procedure for selective patients [1]. However, the laparoscopic hepatectomy needs longer learning time and more costs of surgical instruments. In addition, surgeons need to create a larger wound to remove the specimen at the end of the laparoscopic hepatectomy. Hirokawa [2] had reported small right subcostal incisional left hepatectomy. In this report, we present our experience of small-incision open hepatectomy with concept of minimally invasive surgery for tumors at the left lateral segment (LLS) of the liver. The methods of inflow and outflow control during parenchymal transection were described in detail.

## Material and methods

We designed a study to assess the potential benefits, safeness, and feasibility of LLS hepatectomy through a small midline incision, hanging maneuver, with or without Pringle maneuver. Between November 2010 and July 2011, hepatectomies through small incision for nine patients with benign and malignant tumors were performed at the Kaohsiung Chang Gung Memorial Hospital, Taiwan. All lesions were located in segments 2 and 3 in well-compensated cirrhotic patients (Child-Pugh class A). Preoperative evaluations were as that mentioned in our previous published article [3]. The operation time, incisional width, operation outcome, hospital stay, hospital cost, and instrument cost were studied. The statistic method was Student’s *t* test. The preoperative demography is presented in Table 1. The institutional review board of the Kaohsiung Chang Gung Memorial Hospital in Taiwan approved this study (104-5244B).

**Table 1:** Preoperative demography

No	Age (y/o)	Sex	BMI (kg/m^2^)	Underline diseases	Cirrhosis	Tumor size (cm)	ICG	AFP
1	73	M	20.2	HBV	+	2.2	10.74	3.81
2	59	M	25.0	HBV	−	11	4.3	172.23
3	72	M	25.4	HBV	+	4.5	7.8	3
4	61	M	26.7	-	−	6	1.96	-
5	40	F	32.3	HBV	+	3.5	7.5	6.56
6	49	M	24.6	HBV, HCV	+	2	7.5	4.8
7	74	M	23.6	-	−	2	-	3.97
8	58	F	23.9	HBV	+	6	2.89	2.31
9	46	M	27.7	HBV	+	3	3.7	31.27
Average	59.11		25.5			4.47	5.80	89.74

*BMI* body mass index, *HBV* hepatitis B virus, *HCV* hepatitis C virus, *ICG* indocyanine green, *AFP* alpha-fetoprotein

## Technique

**Fig 1:**
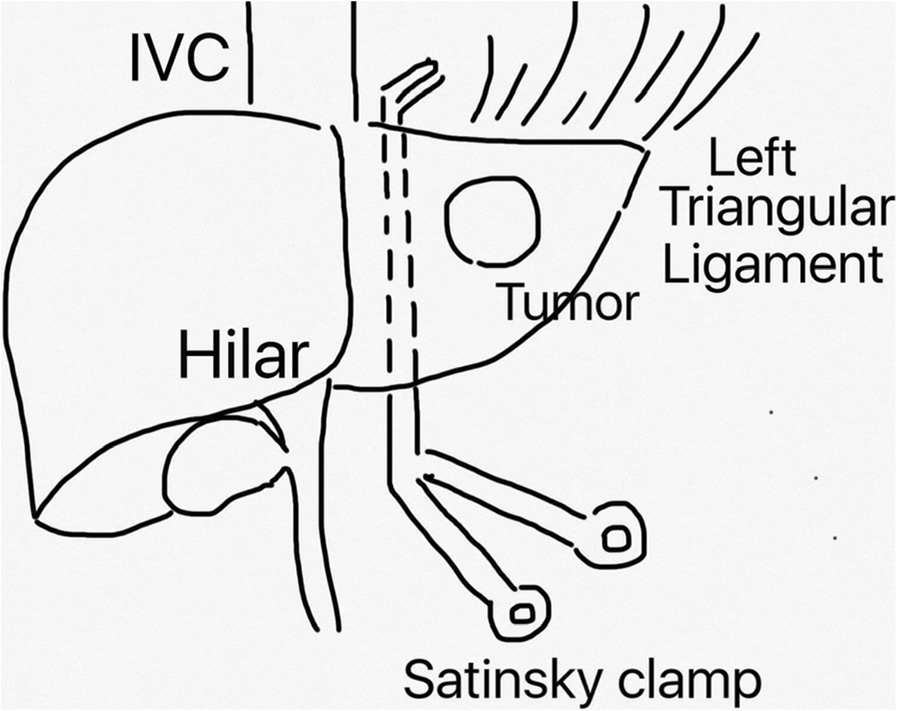
The sketch graph of Satinsky position

**Fig 2:**
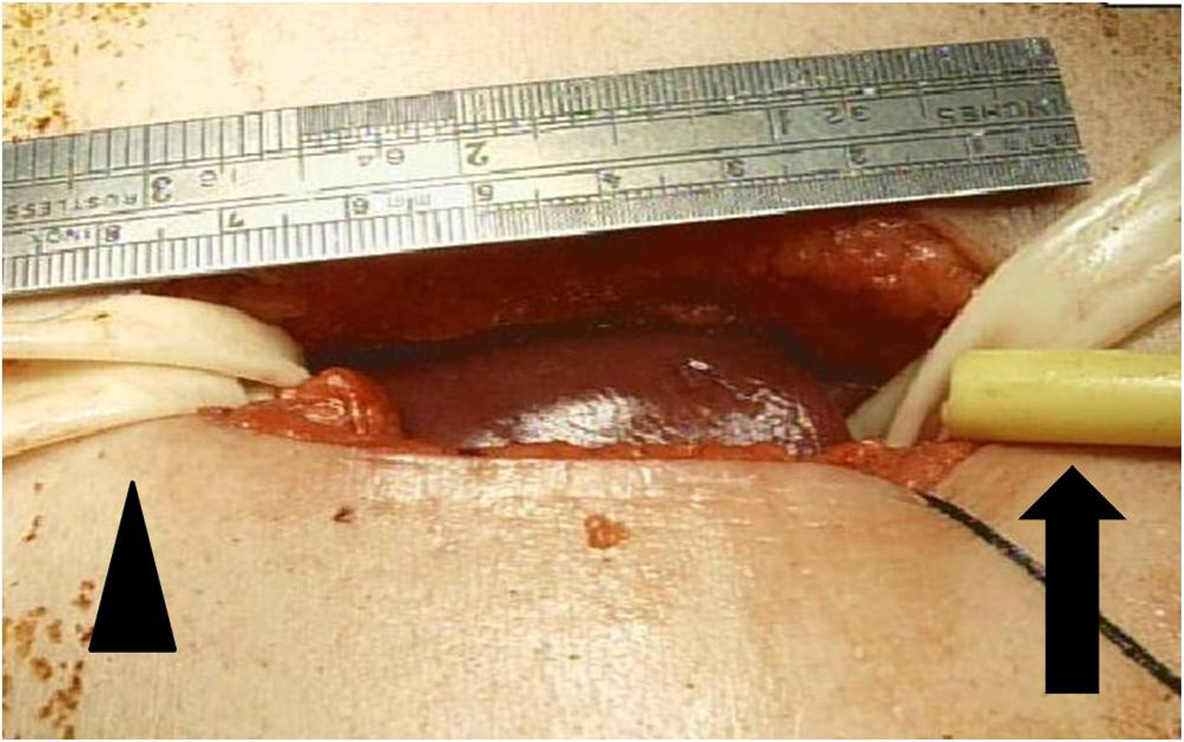
Pringle maneuver with Rumel tourniquet (*black arrow*) and outflow control with hanging maneuver (*black arrowhead*)

The patient is placed in a supine position; a nasogastric tube is inserted to facilitate gastric decompression. The abdomen is opened through an 8-cm upper midline incision, just below the xiphoid process. Under temporary retraction by an assistant, the ligamentum teres is ligated and divided, and the falciform ligament is incised and separated from the anterior abdominal wall. A firm ligature is taken on the ligamentum teres, which acts as a useful retractor during subsequent dissection. The abdominal wound is kept widely open by a self-retaining retractor that also plays a role in elevating the rib cage cephalad for better exposure. The falciform ligament is then divided along the anterior surface of the liver as far back as the suprahepatic inferior vena cava (IVC). Then, duplex ultrasound is used to evaluate the liver tumor and determine the transection line to get adequate resection margin. Just at left side of left hepatic vein (LHV), we divided the left triangular ligament to create a small orifice with width of 2 cm for further liver retraction. It is important to mention that the left triangular ligament is not totally dissected at this moment. After encircling the hepatogastric ligament, a Satinsky clamp is passed behind the left liver cranially with great care toward the orifice on the left border of the LHV (Fig. 1). A hanging tape, seized with the clamp, is used to carefully pull the liver toward the anterior direction. Then, Pringle maneuver is applied by using a special tape (Rumel tourniquet) around the hepatoduodenal ligament for inflow control (Fig. 2). During parenchymal transection, the patient is placed to an approximately 15° reverse Trendelenberg position with central venous pressure maintained less than 5 mmHg. Parenchymal transection is started at the anterocaudate direction of the liver, and the transection line was along the left border of the falciform ligament. Cavitron ultrasonic surgical aspirator (CUSA; Valleylab, Boulder, CO, USA) and bipolar forceps were used for parenchymal transection. The hanging maneuver is done more tightly toward the anterior direction to surround the transaction plan and allow outflow occlusion during transaction of the liver. The remaining parenchymal transection continues cephaladly and posteriorly with left hepatic vein division and suture ligation. Then, the left triangular ligament is dissected and the liver is extracted with a special bag (Taisox LDPE Film Grade Polymer 6334F) to avoid wound contamination. The abdominal incision is closed layer by layer.

## Results

The standard open technique was successfully performed in all nine patients. The Pringle maneuver was applied in six patients during the parenchymal transection. The mean operation time was 219.9 ± 44.8 min. The mean transection time was 74.5 ± 51.7 min. The mean blood loss was 77.2 ± 58.2 ml. No patients required intraoperative or perioperative blood transfusion. The mean incisional width was 8 ± 0.9 cm. The perioperative results are listed in Table 2. The pathologic examination revealed seven hepatocellular carcinoma (HCC), one hemangioma, and one focal nodular hyperplasia. The mean section margin was 19.7 ± 16.5 mm. No patient had surgical complication. The postoperative stay was 6 ± 1.6 days. All patients recovered and returned to normal walk between 4–6 days. Compared with traditional open surgery, our series has shorter mean postoperative hospital stay (6 vs 10.5 days).

**Table 2:** Perioperative and postoperative result

No	Pringle maneuver	Op time (min)	TT (min)	BL (ml)	Incisional width (cm)
1	−	233	199	100	7
2	−	264	88	100	7
3	−	306	100	200	10
4	+	208	55	50	8
5	+	196	54	100	8
6	+	221	44	5	8
7	+	157	31	30	8
8	+	177	40	30	8
9	+	217	60	80	8
Average		219	65.67	77.2	8

*OP time* operation time, *TT* transection time, *BL* blood loss

The mean hospital cost (US$ 4895.6 ± 846.3) was also lower than patients who receive traditional open hepatectomy (US$ 6258). But it does not achieve statistical significance. Our hospital cost was much cheaper than that in the other countries and series (US$ 4,895 vs US$ 15,104) [4]. The postoperative results are listed in Table 3.

**Table 3:** Postoperative outcome

No	Pathology	Section margin (mm)	Comp	POS (day)	Hospital cost (US$)	Surgical instrument cost (US$)
1	HCC	60	Nil	5	4976.7	451
2	HCC	10	Nil	7	5445.9	321
3	HCC	10	Nil	7	6496.5	321
4	Hema	5	Nil	8	4569.3	321
5	HCC	10	Nil	4	4646.8	321
6	HCC	15	Nil	4	5325.0	321
7	FNH	25	Nil	5	3397.5	321
8	HCC	22	Nil	8	4810.2	321
9	HCC	20	Nil	6	4392.4	321
Average		19.67		6	4895.6	335.4

*HCC* hepatocellular carcinoma, *Hema* hemangioma, *FNH* focal nodular hyperplasia, *SM* section margin, *Comp* complication, *POS* postoperative hospital stay

We compared the outcome of our series with laparoscopic LLS segmentectomy in literature (Table 4) [5]. The two series have similar cirrhosis background, benign and malignant tumor lesions, and tumor size. Our series applied Pringle maneuver in most patient and had less blood loss compared with literature. Besides, our hospital cost (4896 ± 798 vs 8962 ± 943) and instrument cost (335 ± 40 vs 2138 ± 381) was much cheaper.

**Table 4:** Compared with laparoscopic left lateral segmentectomy in literature

	Our series	Laparoscopic left lateral segmentectomy in literature
Age (years) mean ± SD	59 ± 11	51 ± 6
Sex (M/F)	7/2	15/18
Benign/malignant	2/7	5/28
Cirrhosis/normal	6/3	23/10
Child-Pugh class (A/B)	9/0	20/3
Tumor size (mm) mean ± SD	45 ± 27	46 ± 10
Operation time (min)	219 ± 42	151 ± 32
Blood loss (ml)	77.2 ± 54.8	173.3 ± 131.1
Use of Pringle maneuver (%)	66.7 %	9.1 %
Postoperative stay (days)	6.0 ± 1.5	3.6 ± 1.0
Complication (%)	0	9.1
Hospital costs ($)	4896 ± 798	8962 ± 943
Surgical instrument cost ($)	335 ± 40	2138 ± 381

*SD* standard deviation

## Discussion

According to literatures, laparoscopic approach to LLS hepatectomy should be considered a standard practice [6, 7]. The benefits of the procedure were small wound, fewer blood loss, short hospital stay, better postoperative life quality, and quick return to activity [8, 9]. Besides, the incidence of incisional hernia was lower compared to that of open surgery [10]. But the disadvantages of laparoscopic surgery were the high expense, the need of learning curve, the lack of three-dimensional visualization, the absence of gentle and safe laparoscopic retracting devices, the lack of tactile feedback [11], the difficulty to control bleeding, oncological risks including the doubtful ability to perform oncological resections, and the potential for tumor cell seeding through surgical ports [8, 12]. Laparoscopic segmental and sectional resections can be more technically demanding than traditional hepatic resections because these are often performed without inflow control. In addition, surgeons may need to extend [13] or create [14] a new abdominal wound to extract the liver with tumor at the final procedure of the operation.

Because of the above reasons, many surgeons reported alternative operations for liver tumor resection under minimally invasive concept such as hand-assisted laparoscopic hepatectomy or laparoscopic-assisted open hepatectomy. Hand-assisted technique during these laparoscopic procedures can afford several benefits that include the ability to use the surgeon’s hand to help stabilize and mobilize the liver and, in cases of hemorrhage, the use of temporary digital control by the direct application of pressure [15, 16]. The HALS study group [11] and the Southern Surgeons’ Club Study Group [17] have concluded that the hand-assisted laparoscopic technique is a useful and feasible alternative for the management of the cases that are too complex or time-consuming to be managed by purely the laparoscopic approach. Koffron et al. reported hybrid method with laparoscopic mobilization of the target liver lobe, followed by standard open liver resection through the small midline incision [18]. Nitta et al. emphasized the hanging technique in laparoscopic-assisted open hepatectomy through small right subcostal incision [19]. Laparoscopy-assisted hepatectomy opens new possibilities for combination laparoscopic techniques with those which are used during open procedures and allows easy extraction of tumor through the small incision [20].

One of the very useful and now widely accepted techniques for both open and laparoscopic hepatic resections is the hanging maneuver [21]. This maneuver was first reported by Belghiti and created in the anterior approach hepatectomy [22]. The hanging maneuver is an important advancement in liver surgery technique and consists in the creation of a tunnel between the anterior surface of the IVC and the liver parenchyma to avoid liver rotation, to reduce liver manipulation potentially responsible for lower tumor cell dissemination, and to provide better exposure and hemostasis of the deeper section plane together with IVC protection. In our procedures, we use the hanging maneuver in all nine patients to promote parenchymal resection through a small laparotomy wound smoothly.

Pringle maneuver is usually applied during liver resection for occluding inflow and reducing blood loss [23]. In the initial three patients, we did not apply Pringle maneuver because the instrument interrupted the small operation field. But these patients had more blood loss. In the next six patients, we tried different methods for inflow control. Initially, we used Satinsky to clamp the hepatoduodenal ligament as traditional open hepatectomy. But the instrument interrupted the small operation field. Rumel tourniquet with a special tape was an alternative method and gave a wider safety margin for patients with chronic liver disease and compromised hepatic reserve by causing less ischemia-reperfusion injury to the remnant liver [24]. In our series, six patients who received Pringle maneuver had fewer blood loss compared with the three patients without Pringle maneuver (*P* = 0.232) (Mann-Whitney test).

Although laparoscopic hepatectomy has many benefits, there should be more consideration for patients and countries with low economic level. In a critical financial time for the health system in almost all developing countries, a cost-effectiveness consideration is key issue [8]. In 2011, Hirokawa [2] had reported small right subcostal incisional left hepatectomy which achieves surgical safety and minimum invasiveness simultaneously. In this report, we have presented our procedure of small midline incisional hepatectomy for tumors located at LLS. The key points of this technique are smaller midline incision (8 cm), no preliminary dissection of left triangular ligament before parenchymal transaction that allows no touch technique, hanging maneuver without flow occlusion, Pringle maneuver that allows inflow control, easy extraction of liver even with big tumor, and no expensive laparoscopic instruments. The overall outcome of our series seems similar to traditional open or laparoscopic LLS hepatectomy, but our method still has some advantage for the patient and surgeon. According to previous reports, most surgeons emphasize shorter hospital stay and lower hospital cost of patients who receive laparoscopic hepatectomy. In Taiwan, the national health insurance covers most hospital costs including ward expense. Patients do not need to pay for ordinary ward expense if they were admitted in a room for three patients. They pay only US$50 daily if they live in rooms for two patients or US$100 daily if they live in a single room. So the cost of an ordinary ward is cheap, and it rarely increases a patient’s burden. On the contrary, the national health insurance did not cover expensive operative procedures such as laparoscopy. Most patients hesitate to accept laparoscopic surgery because of the costs. Furthermore, in a patient who cannot afford CUSA, parenchymal transection with fracture technique will be used. As a result, we compared the hospital cost and instrument cost with literatures. We know it is difficult to compare the hospital cost between different countries with different economic levels. They can be the cohort comparison with the published data.

In addition of low expenditure and shorter postoperative hospital stay, this technique is feasible in most centers not currently performing the pure laparoscopic technique. It can be a transition from open hepatectomy to laparoscopic hepatectomy. Table 5 showed the evolution of minimally invasive LLS hepatectomy. Future directions should include prospective randomized trials with particular focus on LLS hepatectomy, long-term outcomes, and dissemination of the surgical technique.

**Table 5:** Evolution of minimally invasive hepatectomy

	Remark		
Traditional open LLS hepatectomy	Well established	Standard	Larger wound
Laparoscopic LLS hepatectomy	1996	Minimal invasive	Long learning curve
			High economic cost
Laparoscopic assisted or Hand assisted laparoscopic LLS hepatectomy	1999	Improved mobilization during laparoscope	Long learning curve
			High economic cost
Small-incision open LLS hepatectomy	2012	Low economic cost	Smaller wound
		Short learning time	

*LLS* left lateral segment

## Conclusion

Combination of small incision, hanging maneuver, and Pringle maneuver could be applied for resection of the LLS of the liver in some group of patients. This method achieves the concepts of minimally invasive surgery, oncologic resection of tumor, lower cost, and shorter hospital stay.

## Abbreviation

AFP: alpha-fetoprotein
HCC: hepatocellular carcinoma
ICG: indocyanine green
IVC: inferior vena cava
LHV: left hepatic vein
LLS: left lateral segment
